# Case Report: En bloc resection of inferior vena cava and renal cell carcinoma with level IV tumor thrombus associated with tumor thrombus embolization to the pulmonary arteries: presence of blood vessels inside the tumor thrombus

**DOI:** 10.3389/fonc.2025.1511980

**Published:** 2025-09-17

**Authors:** Juan Dugarte, Daniel H. Buitrago, Ramona Nicolau-Raducu, Joshua P. Raber, Hugo Kaneku, Angel Alvarez, Gaetano Ciancio

**Affiliations:** ^1^ Department of Surgery, University of Miami Miller School of Medicine, Jackson Memorial Hospital, Miami, FL, United States; ^2^ Miami Transplant Institute, University of Miami Miller School of Medicine, Jackson Memorial Hospital, Miami, FL, United States; ^3^ Department of Anesthesiology, Solid Organ Transplant & Vascular Anesthesia, University of Miami School of Medicine, Miami FL, United States; ^4^ Department of Urology, University of Miami Miller School of Medicine, Jackson Memorial Hospital, Miami, FL, United States

**Keywords:** renal cell carcinoma, level IV tumor thrombus, en bloc resection, transesophageal echocardiography, cardiopulmonary bypass, tumor thrombus

## Abstract

Renal cell carcinoma (RCC) is an aggressive kidney cancer often diagnosed at an advanced stage. This type of kidney tumor can be associated with tumor thrombus (TT), which can extend into the inferior vena cava (IVC) and, in severe cases, into the right side of the heart. Managing RCC with TT is particularly complex when extension of the TT into the right heart is present, as the use of intraoperative transesophageal echocardiography (TEE) and cardiopulmonary bypass (CPB) aid in performing a complete TT surgical resection. In terms of tumor neo-vessels, solid tumors such as RCC-TT depend on a vascularized connective tissue stroma for growth, proliferation and malformation which are supported by various factors promoting these processes. Herein, we present the case of a 69-year-old patient with a right renal tumor with a TT extending through the IVC up into the right side of the heart. During the surgery, a segment of the TT embolized into the pulmonary arteries, highlighting the surgical challenges, the use of CPB, and TEE in managing such cases, as well as discussing the implications of finding blood vessels inside the TT.

## Introduction

Renal cell carcinoma (RCC) affects approximately 400,000 people annually, causing nearly 175,000 deaths, with incidence expected to rise (1). A study from Leiden University found that 13.3% of 647 RCC patients had a tumor thrombus at diagnosis (2). Additionally, resection of RCC with tumor thrombus (TT) extending into the inferior vena cava (IVC), particularly when it involves the retrohepatic segment, extends above the diaphragm, or reaches the right atrium remains surgically challenging. However, five-year survival rates can approach 50% with complete surgical resection (3). Current techniques for resecting supradiaphragmatic RCC with TT often involve cardio-pulmonary bypass (CPB), with or without deep hypothermic circulatory arrest, although this approach has been associated with coagulopathy and renal failure (4–6). Despite the undesired consequences of its use, some authors support using CPB in order to reduce massive bleeding and ensure that a complete resection is performed (7–11).

Anesthesia monitoring using transesophageal echocardiography (TEE), plays a crucial role in both guiding the surgical resection and maintaining hemodynamic stability throughout the procedure (12). It also ensures that no tumor thrombus emboli are present in the pulmonary arteries or that no thrombus extends into the right atrium (RA) (13). Recently, in terms of the surgical approach, organ transplant-based techniques are employed, with the primary method being piggy-back liver mobilization to fully expose the retrohepatic IVC, with the goal to avoid the use CPB or veno-venous bypass (in as many cases as possible) (14).

A major driver of RCC venous progression with tumor thrombus formation is angiogenesis. This process fuels tumor proliferation and growth, primarily through the cytokine VEGF-A^164/5^ (Vascular Endothelial Growth Factor Type-A^164/5^), which enhances vascular permeability, stimulates endothelial cell migration and survival, plays a role in apoptosis, and reprograms gene expression in endothelial cells (15, 16).

We present here a patient with RCC with Level IV TT extending into the right atrium. The patient underwent right radical nephrectomy with removal of the TT en bloc with the IVC, but during the liver mobilization of the IVC, a segment of the TT embolized to the pulmonary arteries. This catastrophic event highlights the complexity of managing advanced RCC, having CPB standby to avoid the occurrence of a severe adverse event, and emphasizes the importance of a multidisciplinary approach and advanced surgical techniques to ensure successful outcomes.

Notably, the presence of blood vessels within the tumor thrombus has been demonstrated for the first time. This vascularization may explain the necrosis observed within segments of the tumor thrombus, particularly when there is an imbalance between the rapid growth of the tumor thrombus extending into the IVC and the angiogenesis process.

## Case presentation

**Figure 1:**
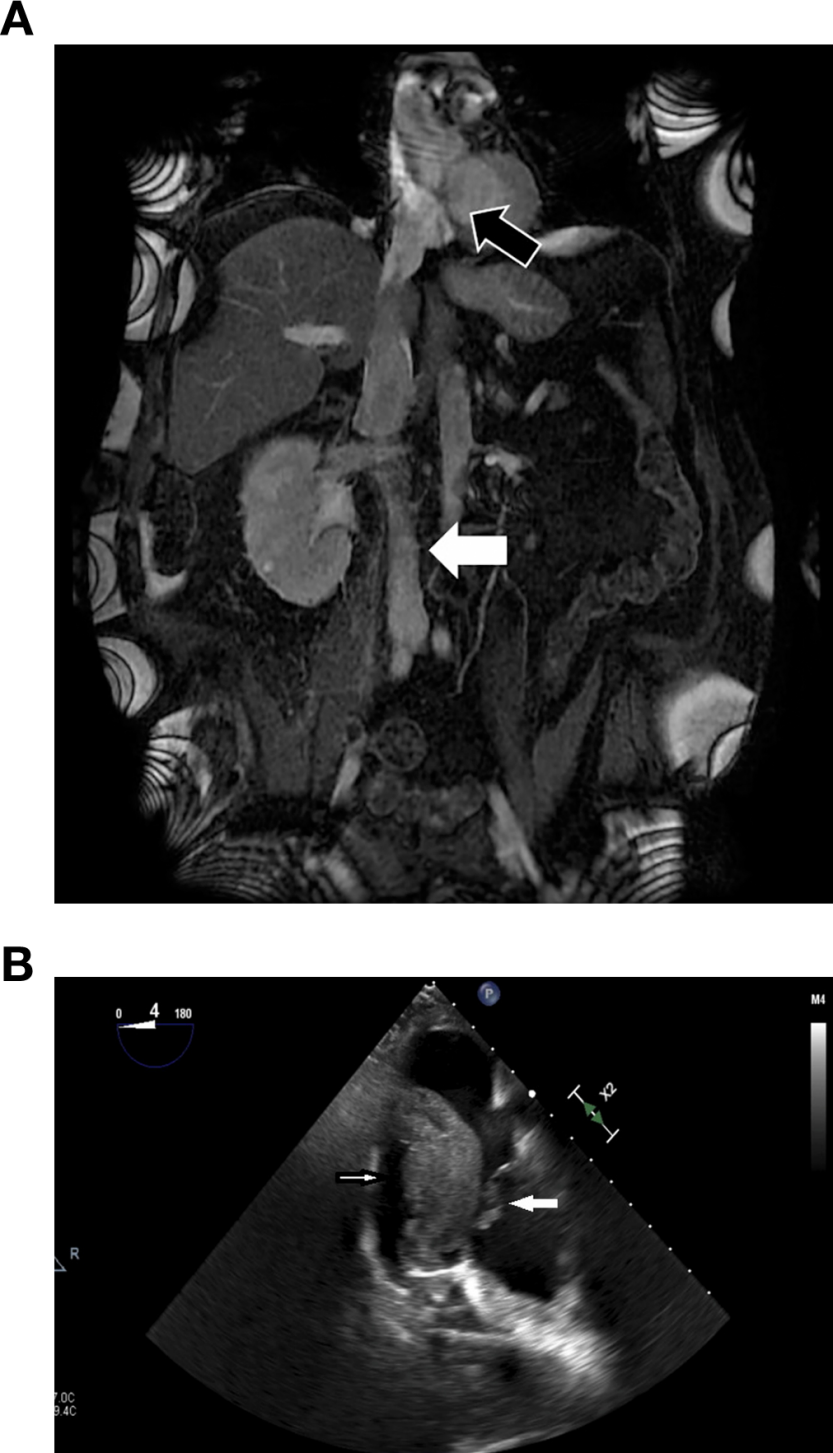
**(A)** Magnetic resonance imaging showing a right kidney tumor and the inferior vena cava (IVC) full of tumor thrombus (TT) all the way to the right atrium (black arrow) and blood thrombus below the TT (white arrow). **(B)** Four-chamber transesophageal echocardiography view showed a segment of TT migrated to RA (small white arrow) and bouncing on tricuspid valve (big white arrow).

A 69-year-old male with a history of hypertension. Complaining of back pain and lower extremity edema, routine blood work revealed a creatinine level of 1.4 mg/dL, and further investigation with ultrasound identified a right renal mass. Abdominal magnetic resonance imaging (MRI) (**Figure 1A**) confirmed a right renal tumor measuring 14 cm in diameter with TT extending through the IVC above the diaphragm into the right atrium, classified as level IV according to the Neves and Zincke classification system (17).

The patient denied any previous episodes of fever, hematuria, dysuria, or any other lower urinary tract symptoms. Computed tomography (CT) scan of the chest and abdomen showed no metastases; therefore, complete surgical excision of this case of RCC with level IV TT was the recommended surgical option. This case report is in accordance with the University of Miami Institutional Review Board and Helsinki Declaration (as revised in 2013). The patient was informed of the risks of surgery, including infection, bleeding, blood transfusions, pulmonary emboli, and impossibility of complete surgical excision. Written informed consent was obtained prior to surgery.

## Procedure in detail

A modified Chevron incision was performed approximately 2 fingerbreadths below the right costal margin and extended out laterally to the mid-axillary line and medially 3-4cm towards the left costal margin. A Thompson retractor was used to elevate the costal margins. The right kidney with the tumor was dissected laterally and posteriorly, and then mobilized medially. The renal artery was posteriorly identified, ligated and divided (18), causing the collateral circulation to collapse, minimizing blood loss. Subsequently, liver mobilization was performed using the Piggy-back liver transplant technique (19), with ligation of the ligamentum teres, falciform ligament and left triangular ligament. The liver was mobilized off the IVC, and small hepatic veins were ligated and divided to expose the infrahepatic, intrahepatic and suprahepatic portions of the IVC (14). However, after the division of the IVC ligament, a segment of TT floated free into the right atrium (**Figure 1B**), then moved into the right ventricle and finally lodged in the pulmonary arteries (PAs). The cardiothoracic team was in the operating room and proceeded with the CPB. Median sternotomy was performed, and pericardium was opened. After administration of a total 35,000U heparin, central cannulation was performed via a 20Fr Opti arterial cannula in the ascending aorta and a 24Fr right angle metallic tip cannula into the superior vena cava. Cannulation onto the inferior vena cava was avoided due to the presence of tumor within centimeters below the atrio-caval junction. Caval tapes were passed around both cavae. Partial CPB was initiated once activated clotting time was above 480 and temperature drifted to 36 °C. Both Cavae were snared. A 4–0 Prolene stay sutures were placed on the anterior surface of the main PA trunk. The PA was then opened longitudinally using an #11 blade scalpel. The stay sutures were clipped to the drape to open the lumen, and a drop sucker was placed into the main PA to aid in exposure and visualization. A large tumor thrombus was seen sitting at the PA bifurcation. The tumor was friable and it was removed by gentle traction using ring forceps, preserving its integrity. Further exploration into the right and left main PA did not reveal the presence of any intraluminal residual tumor. No TT was left in any main branch nor in the right sided chambers. The main PA was closed with 4–0 Prolene, running suture, two layers. When rewarming was completed, the patient was weaned off the CPB. Total bypass time was 59 minutes. Venous decannulation was performed. Protamine was administered. The aortic cannula was removed. Cannulation sites were revised for hemostasis and reinforced. Hemostasis of the entire mediastinum was performed. One mediastinal 28Fr chest tube was placed.

After the removal of the embolized segment of the TT and reversing the coagulopathy, the TT was removed en bloc along with the right kidney and the IVC (**Supplementary Figure**). The IVC was stapled below the major hepatic veins, left renal vein, and 2 cm above the IVC bifurcation. Stapling the IVC prevented postoperative bland thrombus (BT) embolism (**Figure 1A**). At the end of the surgery, a final TEE was performed to ensure no pulmonary artery emboli or TT were present. The mediastinum was packed, because the patient was coagulopathic, and the chest was partially closed. A total of 28 units packed red blood cell, 11 units fresh frozen plasma, 4 units platelets, 500 units prothrombin complex and 2g fibrinogen concentrate was administered to achieve hemostasis and hemodynamic stability. The definite chest closure was done 48 hours after the surgery without complications.

Pathology examination revealed a right kidney mass and IVC as a 1020-gram total nephrectomy specimen, measuring 19 × 12 × 11 cm. Attached to the kidney was a segment of the IVC, 10 cm in length and 4 cm in diameter, which was 100% occluded by a thrombus. The tumor measured 14 × 10 × 7.4 cm. The mass was confined within the renal capsule and Gerota’s fascia. It was a clear cell RCC Fuhrman grade 4, and lymph nodes were negative for carcinoma. AJCC (8th Edition) Classification was pT3cNxMn/a (20). The IVC TT was attached to the intima but did not appear to involve either of the surgical resection margins.

**Figure 2:**
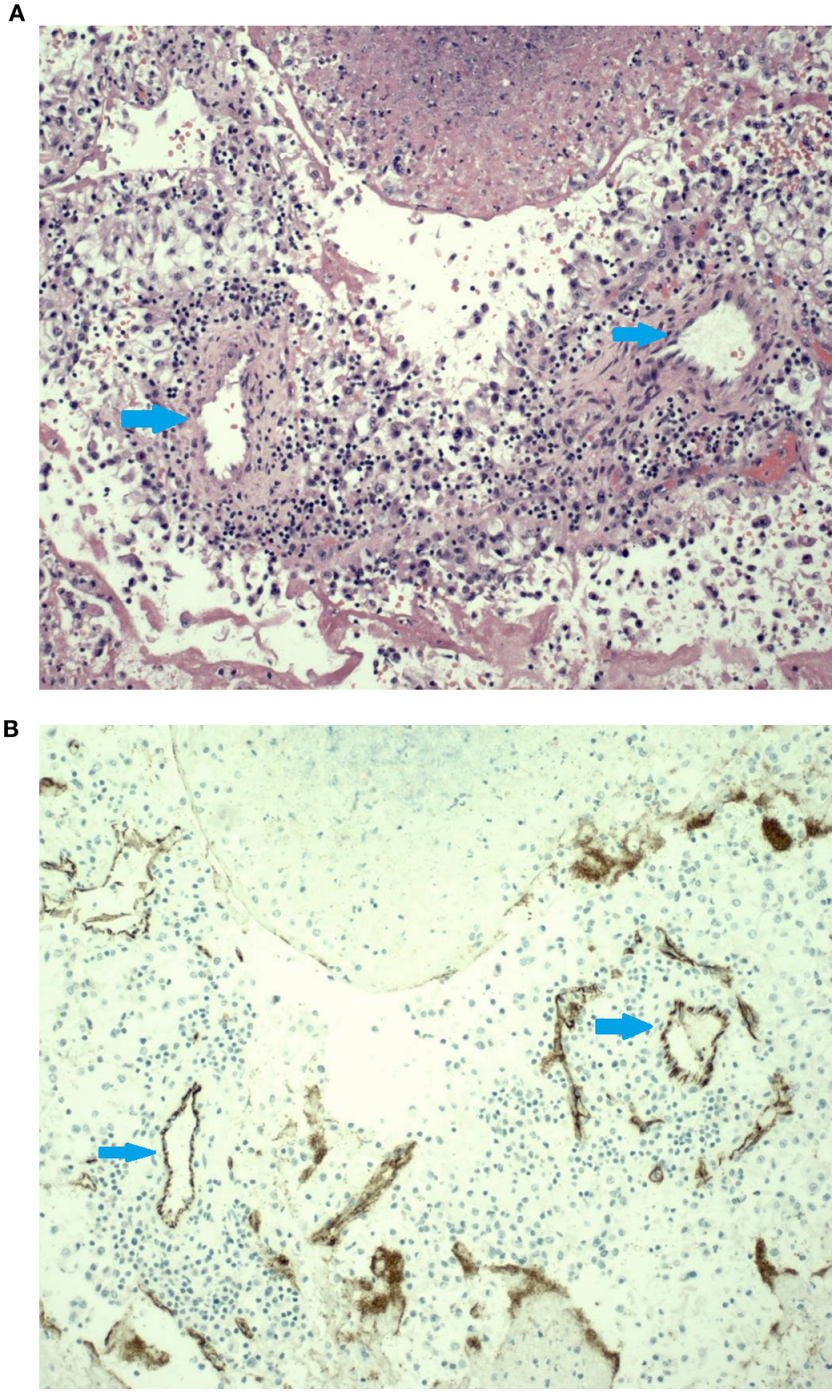
**(A).** The hematoxylin & eosin (H&E) slide shows sections of small caliber arteries surrounded by a mild mononuclear inflammatory infiltrate surrounded by areas of tumor necrosis. Histologically, these small arteries do not look like the thin-walled vessels typically seen in this type of tumor. **(B)** Immunohistochemical stain for CD31 highlights the endothelial cell lining on these small arteries. Blue arrow showed the vessels.

The TT that embolized to the pulmonary artery was stained with Hematoxylin-eosin (**Figure 2A**), showing clear cell renal cell carcinoma with rhabdoid features, as well as small caliber vessels. In addition, this specimen was noted to be 50% necrotic. The same section of tissue was used for immunohistochemical (IHC) staining (**Figure 2B**) to evaluate CD31, a marker for endothelial cells, effectively demonstrating the presence of blood vessels within the tumor thrombus.

The patient was discharged home on post-operative day 20. The patient had an uneventful recovery showing a creatinine level of 1.5 mg/dL at 12-months of follow-up, he was started on combination therapy with pembrolizumab and axitinib and remains cancer-free.

## Discussion

RCC represents 80%–85% of all primary renal neoplasms (21) and ranks as the ninth most common cancer in the United States (22). Studies indicate that the likelihood of TT being present in diagnosed RCC is approximately 13% (2). This surgical case described here highlights a critical intraoperative event that can occur during resection of RCC with TT: embolization of the TT to the pulmonary arteries. In our case, this complication occurred following division of the IVC ligament, an event we have encountered in previous cases as well (23) but it may also occur following manipulation of the IVC (24).

The classic triad of renal cell carcinoma (RCC), flank pain, hematuria, and a palpable abdominal mass is now rarely observed. Currently, more than 70% of RCC cases are detected incidentally through noninvasive imaging performed for unrelated or nonspecific symptoms (25). In this case, imaging studies included an ultrasound, which identified a right renal mass; MRI, which revealed a 14 cm right renal tumor with tumor thrombus (TT); and a CT scan, which showed no evidence of metastasis.

The surgery for large RCC, particularly with the existence of a TT occupying the entire IVC, make the procedure complex, challenging and high-risk with innumerable complications like major perioperative blood loss, presence of difficult to reach exposures growing into the right atrium, multiple collaterals and the major risk of developing pulmonary emboli (19). Unfortunately, surgery remains the only viable option to ensure a cure for patients harboring this complex urological tumor (26).

Traditional surgical approaches for resecting supradiaphragmatic RCC tumor thrombus often involve cardiopulmonary bypass, sometimes combined with deep hypothermic circulatory arrest (DHCA) to facilitate the surgery with minimal morbidity to patients (2). However, this technique introduces risks such as coagulopathy and renal failure, which can adversely affect outcomes in this already high-risk surgery (4–6). Over the years, we have developed transplant-based surgical techniques to manage tumor thrombus involving the inferior vena cava, the most critical component of the operation, with the primary goal of avoiding cardiopulmonary bypass (CPB) (8). CPB with or without DHCA is reserved only for rare circumstances, such as the unique clinical situation presented in this case (27).

A multidisciplinary approach was crucial for optimally managing the patient in this case report. Alongside the use of CPB, the anesthesiology team employed TEE to monitor the location and extent of the TT according to the Neves-Zincke classification (17) in real-time.

Similar complex, multidisciplinary surgical approaches combining cardiopulmonary support with TT resection have been described, particularly in patients with concurrent cardiac comorbidities. In such cases, favorable outcomes have been reported, as illustrated by Filomena et al., who documented successful simultaneous management of renal cancer with atrial thrombotic extension and severe chronic coronary artery disease (28).

Stapling of the IVC was successfully performed in this case to achieve its interruption due to blood thrombus (BT) present below the tumor thrombus, which could obscure the resection margins. Stapling the IVC has been shown to effectively manage chronic IVC obstruction, eliminate the need of IVC reconstruction, exert control of the BT, and reduce the risk of embolization during and after surgery (29). BT is a non-tumorous thrombus consisting of platelets, macrophages, and fibrin (30), and its formation is associated with venous stasis or hypercoagulability (31), which are common findings in neoplastic processes like RCC. The coexistence of BT with TT further complicates the diagnosis and surgical management of RCC patients (32).

Alongside the size of the RCC and the extent of TT, the complexity of this surgery is significantly increased by the presence of blood vessels within the TT. One of the main components of neo-vessel formation in TT is VEGF-A^164/5^, a cytokine with exceptionally strong vascular permeability characteristics—50,000 times more potent than histamine. In addition to its role in permeability, VEGF-A^164/5^ also facilitates endothelial cell migration, acts as a survival factor, and helps prevent endothelial cell apoptosis and senescence (16, 33). In addition to VEGF-A^164/5^, which works in tandem with fibrin and stroma in normal wound healing, these components are also significant in TT. While fibrin and stroma are usually regulated and active only briefly in standard wound healing until new vessels form, their activity is markedly overexpressed in TT cases (34). This robust vascular network within TT not only aids in tumor invasion but also complicates surgical procedures. Extensive neo-vascularization enhances thrombus spread and increases the risks of bleeding and embolization. This TT embolization could occur before surgery (35) or during surgery (as observed in the present case), probably due to the presence of necrosis caused by the imbalance of fast growth of the TT inside the IVC and insufficient growth of neo-vessels. The impact of this network of neo-vessels inside the TT on resistance to antiangiogenic therapy remains uncertain. Further analysis of the presence and characteristics of neo-vessels within the TT is needed to evaluate their potential influence on response to chemotherapy.

Finally, the pathology report confirmed that our patient had renal cell carcinoma (RCC) with rhabdoid differentiation, a rare but notably aggressive subtype, seen in approximately 5% of RCC cases. Rhabdoid differentiation is associated with a significantly increased risk of mortality, independent of other established prognostic factors, and is typically indicative of a poor clinical outcome (36). Historically, treatment options for RCC with rhabdoid features have shown limited efficacy. However, recent advances in immunotherapy, specifically immune checkpoint inhibitors targeting PD-1, PD-L1, and CTLA-4 have demonstrated encouraging clinical responses in this subset of patients (37).

Our patient was initiated on combination therapy with pembrolizumab and axitinib. At 12-month follow-up, he remains cancer-free (38).

Overall, the management of patients with renal tumors and associated tumor thrombus is highly complex and should be undertaken at high-volume centers. Referral to such institutions is critical to optimizing outcomes and should be emphasized in this context (39).

In conclusion, the precise assessment of TT extent in RCC, along with the presence of vasculature spread due to neo-vessel formation driven by multiple oncologic factors, is essential for effective surgical planning. This case underscores the critical role of advanced imaging and real-time monitoring with TEE, as well as tailored surgical strategies, such as IVC stapling, to mitigate complications from bland thrombus. Moreover, neo-vascularization within the TT adds significant complexity to the surgical approach. A well-prepared, multidisciplinary surgical team is crucial for achieving optimal outcomes and minimizing complications in complex RCC cases with extensive TT.

## Data Availability

The raw data supporting the conclusions of this article will be made available by the authors, without undue reservation.
