# Supplementary material for: Case Report: En bloc resection of inferior vena cava and renal cell carcinoma with level IV tumor thrombus associated with tumor thrombus embolization to the pulmonary arteries: presence of blood vessels inside the tumor thrombus
Source: Front Oncol. 2025 Sep 17;15:1511980. doi: 10.3389/fonc.2025.1511980 (PMC12484219; doi:10.3389/fonc.2025.1511980)

**Supplementary Figure.** Pathology specimen of the right kidney (black arrow) showing the tumor thrombus inside the IVC (thin white arrow). The right kidney tumor was removed en bloc with the TT inside the IVC, the IVC was stapled below the major hepatic veins, left renal vein and 2 cm above the IVC bifurcation. Also showing the segment of TT that embolized into the pulmonary arteries (blue arrow). RA: right atrium (segmented white arrow).


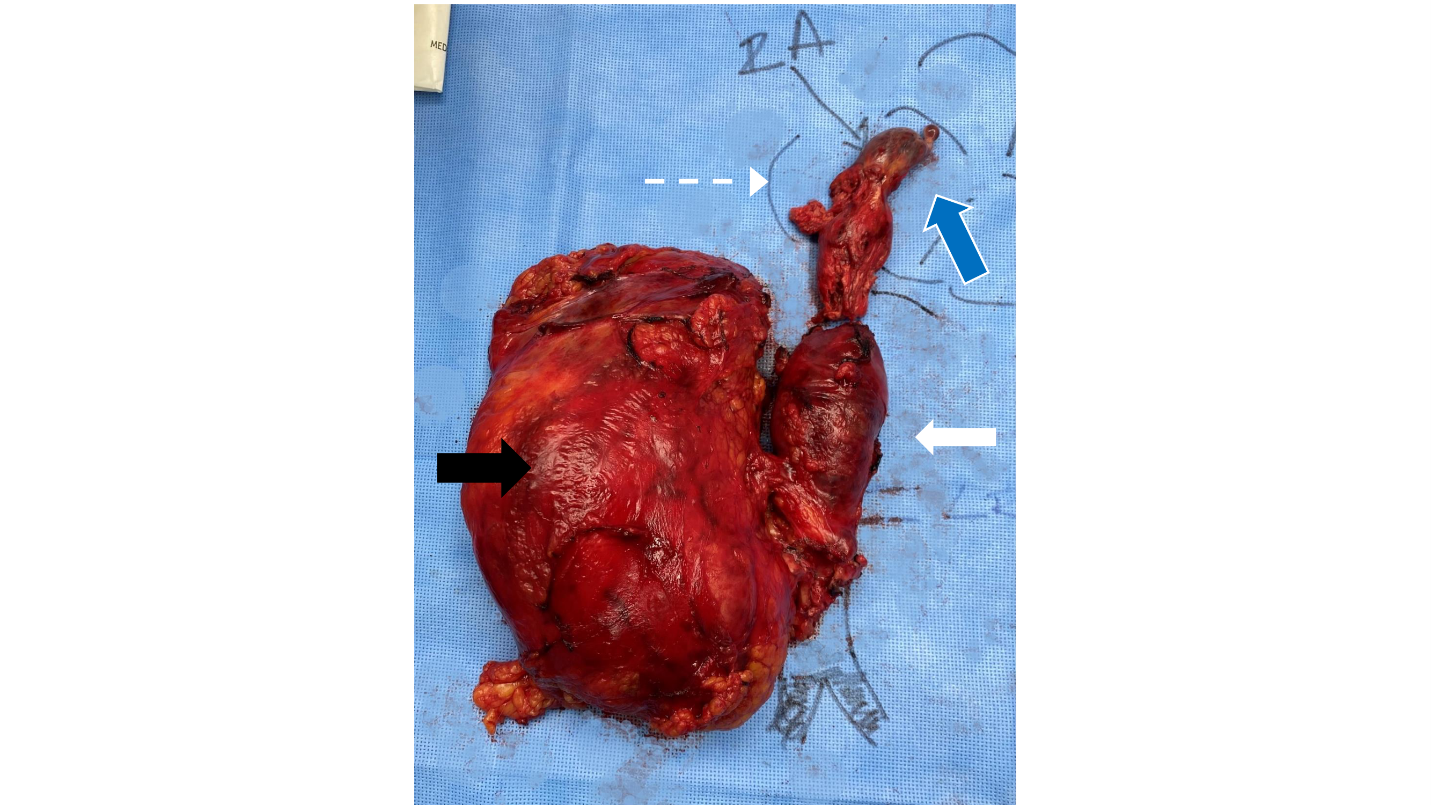

Supplement: Supplementary file 1 [file DataSheet1.docx]
